# On the Crucial Role of Isolated Electronic States in the Thermal Reaction of ReC^+^ with Dihydrogen

**DOI:** 10.1002/anie.202001599

**Published:** 2020-04-06

**Authors:** Jilai Li, Caiyun Geng, Thomas Weiske, Helmut Schwarz

**Affiliations:** ^1^ Institut für Chemie Technische Universität Berlin Straße des 17. Juni 115 10623 Berlin Germany; ^2^ Institute of Theoretical Chemistry Jilin University 130023 Changchun China

**Keywords:** gas-phase reactions, metal carbides, noble metals, rhenium, transition states

## Abstract

Presented here is that isolated, long‐lived electronic states of ReC^+^ serve as the root cause for distinctly different reactivities of this diatomic ion in the thermal activation of dihydrogen. Detailed high‐level quantum chemical calculations support the experimental findings obtained in the highly diluted gas phase using FT‐ICR mass spectrometry. The origin for the existence of these long‐lived excited electronic states and the resulting implications for the varying mechanisms of dihydrogen splitting are addressed.

## Graphical abstract

**As a rare example**, the coexistence of the ground and long‐lived, low‐lying electronic states for the bare ReC^+^ ion is identified. These different electronic states cause distinctly different reactivities towards the thermal activation of dihydrogen.

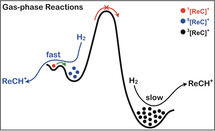

Almost two centuries ago, Döbereiner discovered that a flame is born when dihydrogen flows over finely dispersed platinum in the presence of air.^1^ This observation led to the invention of the lighter named after him, which became quite popular at the time, and prompted Berzelius to introduce the term “catalysis” a couple of years later.^2^ Although platinum as well as other noble metals still are particularly important in technologies requiring highly active catalysts, such as fuel cells,^3^ vehicle emission control equipment,^4^ crude oil refining, or even the conversion of fine chemicals, their usage usually suffers from the serious disadvantage of being quite rare and rather expensive. About 50 years ago, Böhm alleviated the problem by reporting that solid tungsten carbide (WC) exhibits properties similar to those of platinum,^5^ a finding that was confirmed later.^6^ Bare neutral WC is isoelectronic with ReC^+^, and we came across this diatomic cation by the quite unexpected gas‐phase chemistry of cationic 3d‐transition‐metal carbides (TMCs) with methane.^7^ This study prompted us to investigate the reactions of ReC^+^ with dihydrogen.^8^

The concept of using small atomic clusters to serve as models to investigate some aspects of heterogeneous catalysis was proposed decades ago.^9^ In fact, the comparative simplicity of small clusters greatly enhances their value as models and provides an excellent opportunity to investigate mechanistic details of important catalytic processes such as the cleavage of the N_2_ triple bond,^10^ or the industrially relevant coupling of CH_4_ with NH_3_ to produce HCN (DEGUSSA process).^11^ There is consensus that the elucidation of structure–reactivity relationships of prototypical clusters is of eminent importance in a broader scientific context.^12^

The reactivity of clusters in the highly diluted gas phase is mainly governed by two factors: On one hand one may deal with an ensemble of diverse geometric structures,^13^ and, on the other hand, for a given structural isomer different electronic states may exist and play a decisive role.7a, 10a, 14 Unfortunately, the way polyatomic clusters are generated in the gas phase often leads to mixtures of different structural isomers, and in the case of transition‐metal clusters the possibility of multiple, energetically low‐lying electronic states needs to be taken into account as well. In pioneering work performed decades ago, Armentrout, Bowers, and their co‐workers impressively demonstrated the coexistence of multiple electronic states of transition‐metal *atomic* ions.^15^ What about *molecular* ions? Clearly, the simplest conceivable molecular ion consists of just two atoms, and this fact has the additional benefit to exist as one structural isomer only.

Herein we report an unusual observation in the thermal gas‐phase reaction of diatomic ReC^+^ with dihydrogen. After several control experiments aimed at looking for alternative explanations, the results suggest 1) the coexistence of at least two long‐lived, electronic states of ReC^+^, and 2) that these two states differ significantly in their reactivity towards dihydrogen splitting. Mechanistic aspects of the H−H bond activation were elucidated by high‐level quantum chemical (QC) calculations.

ReC^+^ was generated by supersonic expansion of helium into a rhenium/carbon plasma generated by laser ablation/ionization of a disk comprised of compressed rhenium/graphite powder (1:1; molar ratio) using a Nd:YAG laser, operating at 532 nm inside the external cluster‐source of a Fourier transform ion cyclotron resonance (FT‐ICR) mass spectrometer as described previously (for details, see the Supporting Information).^16^ A fraction of the ion population, generated in the external source region, is then guided by a static ion optical system into the ICR‐cell. Next, in a sequence of pulses, argon is admitted to the ICR cell such that the ions collide on average about 1×10^5^ times with argon. This procedure ensures thermalization of hot ions and is usually regarded to quench excited electronic states. After thermalization to room temperature in a first and mass‐selection in a second step the ReC^+^ ions (*m*/*z*=199) were reacted with dihydrogen (H_2_, HD, and D_2_) at a constant pressure and under single collision conditions (for further details, see the Supporting Information and Ref. 14e, 14f, 16). The elementary compositions of the charged particles have been confirmed by exact mass measurements using high‐resolution mass spectrometry.

The FT‐ICR spectrum in Figure 1 a was obtained when only helium was admitted to the ICR cell. Upon leaking H_2_ into the ICR cell at a stationary pressure, a hydrogen‐atom transfer takes place [Eq. (1) 
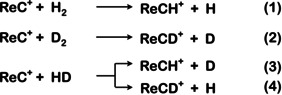
], resulting in the generation of ReCH^+^, signal B in Figure 1 b. The H−H bond scission was confirmed by an isotopic labeling experiment using D_2_ [Eq. (2)]. Here the product ReCD^+^, signal C, is formed, Figure 1 c. In addition, the intramolecular kinetic isotope effect (KIE), derived from the ReC^+^/HD couple amounts to KIE(H/D)=0.43 [Figure 1 d, Eqs. (3) and (4)]. These experimental findings demonstrate that ReC^+^ activates dihydrogen at ambient temperature, and is in contrast to the unligated Re^+^, for which the reaction with H_2_ is endothermic, as described by Armentrout^17^ and confirmed by us by means of FT‐ICR MS. Finally, the absence of a signal for ReCH^+^ in Figure 1 c indicates that reactive background gases RH do not contribute to the consumption of ReC^+^ by hydrogen‐atom abstraction from RH.

**Figure 1:**
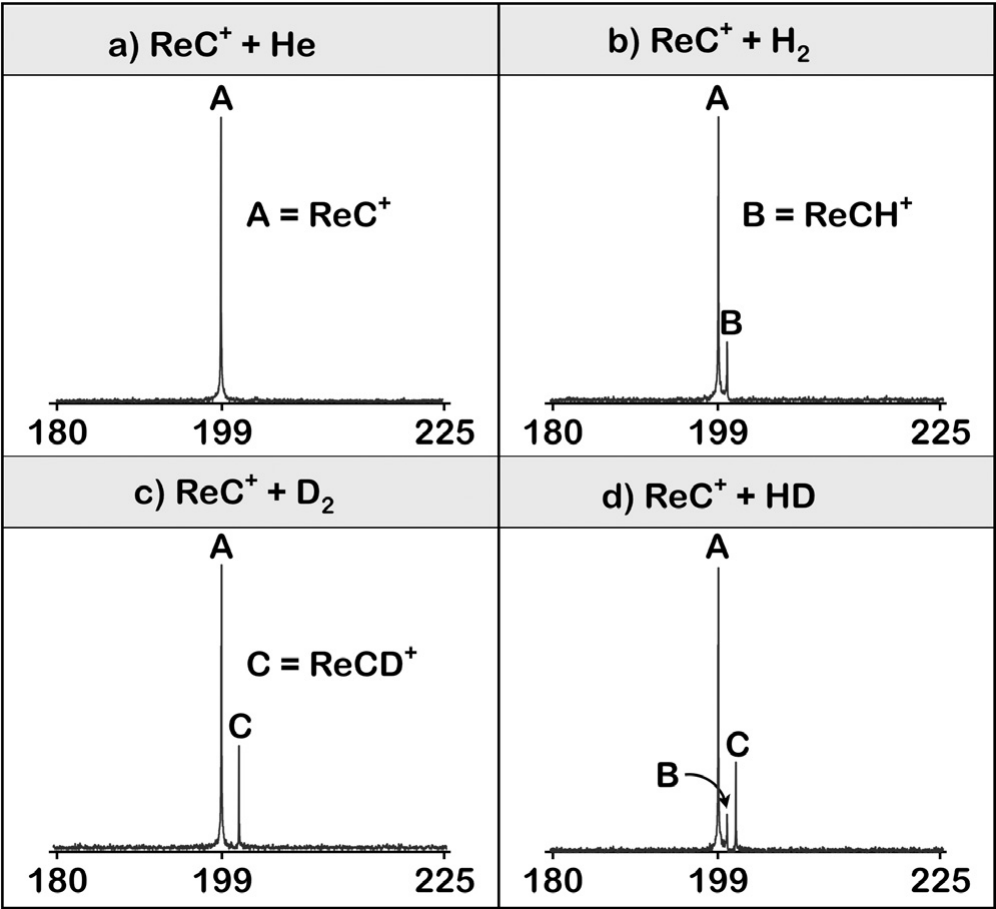
Mass spectra of the thermal reactions of mass‐selected ReC^+^ with: a) He, b) H_2_, c) D_2_, and d) HD at 1×10^−8^ mbar after a time delay of 4 s at ambient temperature. X‐axes are scaled in *m*/*z*, and the *y*‐axes are normalized relative ion abundances.

The reaction kinetics deduced from the time‐dependent change of the abundance of the educt ion, ReC^+^, in the ion/molecule reactions with dihydrogen are illustrated in Figure 2 a. Apparently, the ReC^+^/H_2_ couple exhibits a marked deviation from the strict linearity of a pseudo‐first‐order reaction kinetics; rather, the intensity decline of the charged educt can be fitted very well by the sum of two exponential decay functions, Equation (5), in which *a*_1_ and *a*_2_ represent the relative initial abundances of “different” ions at time *t*=0 and *k*_1_ and *k*_2_ their respective rate constants. A two‐parameter function does not fit the experimental data (for details, see the Supporting Information). Clearly, there exist two regimes in that at the beginning the faster reaction prevails while at longer reaction times the slower process dominates (Figure 2 b).

(5) $$I\left(\mathrm{ReC}{}^{+}\right)\left(t\right)=a{}_{1}\;e{}^{-k{}_{1}\;t}\;+\;a{}_{2}\;e{}^{-k{}_{2}\;t}$$

**Figure 2:**
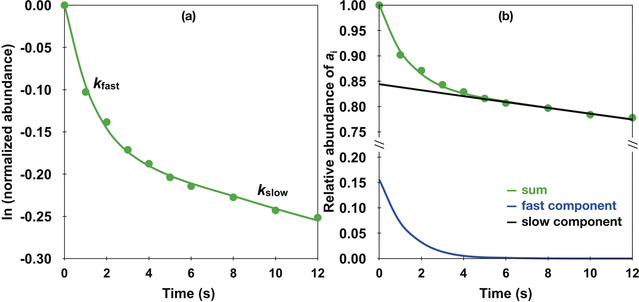
a) Semilogarithmic plot of the ion abundances for the reactions of thermalized ReC^+^ with H_2_ at a pressure of about 1.0×10^−8^ mbar. The full circles represent the measured data points and the curve is a graphical display of the fitting function according to Eq. (5). b) Temporal evolution of the ReC^+^ abundances in the reaction with H_2_ at a pressure of about 1.0×10^−8^ mbar. The green circles are the measured data points, and the green line results by fitting the experimental data using the function given in Equation (5). The blue and black curves display the two separated exponential terms with the blue one representing the reaction of the faster and the black curve that of the slower component.

This two‐component feature persists, irrespective of the degree of thermalization of the precursor ion with argon and the pressure of dihydrogen. Such a situation has been reported for systems in which at least two isomeric species coexist in sizable amounts.13i, 13j, 13k, 13m, 13p, 13r, 13s, 13t Thus, also for ReC^+^, this observation implies the presence of two different species exhibiting different reactivities towards dihydrogen. As other possible factors, for example, the presence of reactive background gases or, in particular, kinetically excited ReC^+^ projectiles,^18^ can be ruled out (Figure 1 c; see Figure S1 in the Supporting Information), the assumption that (at least) two long‐lived, isolated electronic states coexist is not far‐fetched. If true, the co‐existence of a fast and a slow reaction further implies that the mutual transformation of these electronic states is significantly hampered, if not impossible.

Furthermore, the relative abundancies of ReCD^+^/ReCH^+^ vary for the two reactivity regimes when ReC^+^ is reacted with HD. At a pressure of 1.0×10^−8^ mbar and a short reaction time (1 s), where the fast reaction dominates, a value of 1.5 is obtained. At longer reaction times (12 s), at which the slower reaction matters, this ratio increases to 3.1.

The rate‐constant *k*(ReC^+^/H_2_) for the fast component has been determined as 1.7×10^−10^ cm^3^ molecule^−1^ s^−1^, corresponding to a calculated efficiency^19^ of *φ*=11.0 %. For the slower reaction it amounts to only 1.6×10^−12^ cm^3^ molecule^−1^ s^−1^ (*φ*=0.10 %). Owing to the uncertainty in the determination of the absolute H_2_ pressure, an error of ±40 % is associated with these measurements.16b For the relative rate constants, however, the error is much smaller, typically around ±5 %.

The unusual reactivity pattern of ReC^+^ towards dihydrogen poses at least three questions: First, why is it so difficult for the excited state(s) of ReC^+^ to be converted into the ground state? Second, do the different electronic states react the same way with dihydrogen or are there any noticeable mechanistic differences? Third, which of the electronic states of ReC^+^ is responsible for the slow and which for the fast process?

To this end, and to obtain mechanistic insight, high‐level quantum chemical calculations were carried out. According to exploratory diagnostic tests, the ReC^+^/H_2_ system possesses significant multireference (MR) character. Therefore, we followed a protocol that had been successfully applied previously for the theoretical description of MC^+^/CH_4_ couples (M=3d transition metal).7a

Figure 3 displays the potential energy diagrams of the three energetically lowest electronic states of ReC^+^ (see Table S1), together with the crossing points (CPs)^20^ connecting them. Our elaborate calculations at the ZORA‐NEVPT2(10e,10o)/BS3 level of theory, in line with previous CCSD(T)/CBS calculations,^21^ yield a triplet state (^3^Σ^−^) for the ground state of ReC^+^. The positive charge is essentially localized on the metal atom (see Table S2). The lowest excited state corresponds to a quintet state, ^5^Σ^−^, and is by 77 kJ mol^−1^ higher in energy. The second lowest excited state of ReC^+^ is a singlet state, ^1^Γ, and 100 kJ mol^−1^ above the ground state. Additional electronic states of even higher energies are listed in Table S1.

**Figure 3:**
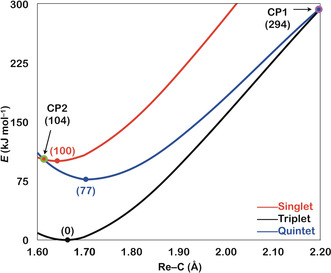
Potential energy diagram (Δ*E* in kJ mol^−1^) of the three lowest electronic states of ReC^+^ including the crossing points as obtained at the ZORA‐NEVPT2(10e,10o)/BS3 level of theory. Ground state (Triplet), black; lowest excited state (Quintet), blue; second‐lowest excited state (Singlet), red. The relative energies of the minima and crossing points (CPs) of the three potential energy curves are given in parenthesis.

Since the potential energy curves of the singlet and the triplet states run almost parallel to each other upon varying the Re−C bond length from 1.6 to 2.2 Å, both states cannot cross in this energy regime. A crossing point, CP1, between the triplet and the quintet states has been located 294 kJ mol^−1^ above the minimum of the triplet surface. Clearly, without substantial energy supply the intersystem crossing (ISC) probability must be extremely low. In addition, the radiative emission of photons from the minimum energy regime of the quintet surface to the ground state triplet is extremely inefficient for the following reasons: 1) the transition from quintet to triplet is spin‐forbidden, 2) the oscillator strength is negligible for this transition owing to the absence of orbital angular momentum for both the ^3^Σ^−^ and ^5^Σ^−^ states; a first‐order spin‐orbit correction is not necessary,^21^ and 3) a qualitative estimation of the lifetimes of this and other low‐lying electronic states reveals that for some of them it can extend into the time regime of seconds and longer (see Tables S6–S9).^22^ In contrast, only 4 kJ mol^−1^ are required for the singlet minimum to pass over to the quintet surface via CP2, and under the given experimental conditions, this ISC is very likely to happen.

A frontier orbital analysis indicates that a decoupling of the paired electrons of the σ(Re‐C) orbital in ^1^[ReC]^+^ is necessary to promote this transition (see Figure S2). Because of the error bar of the QC method used and the way the ions are generated by laser evaporation/ionization, it can be assumed that electronically excited ReC^+^ ions initially are present in both their quintet and singlet states, however, in terms of quantity the ReC^+^ ions in the triplet ground state clearly dominate. Most importantly, while the singlet state could easily cross to the quintet, the latter state cannot be quenched further to the triplet ground state. Obviously, for ReC^+^ there is a coexistence of a long‐lived excited with the electronic ground state.

Next, we consider computationally the reaction mechanisms of the three electronic states of ReC^+^ when exposed to dihydrogen. Simplified two‐dimensional potential energy surfaces (PES's) of the most favorable pathways as well as symmetries and selected structural parameters of key species are shown in Figure 4. An energetically less favored route (see Figure S3) and a frontier orbital analysis (see Figure S4) of the reactions considered in this study are relegated to the Supporting Information.

**Figure 4:**
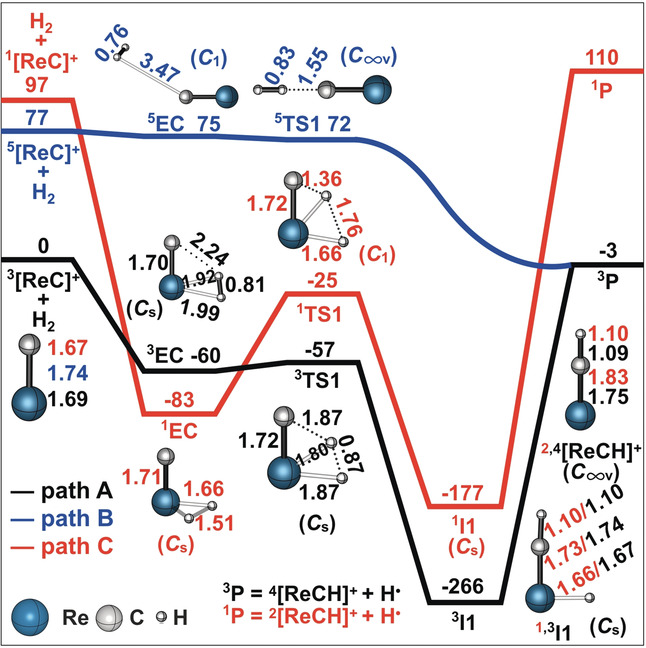
Simplified PES's (Δ*H*_298K_ in kJ mol^−1^) as obtained at the NEVPT2(12e,12o)/BS2//CASSCF(12e,12o)/BS1 level of theory for the reaction of ReC^+^ with H_2_. Key structures with selected geometric parameters and symmetries are also provided. Bond lengths are given in Å. Charges are omitted for the sake of clarity. Relative energies are corrected for possible contributions of ZPVEs.

First the reactivity of the triplet ground state ReC^+^ is considered. According to path A (black) in Figure 4, the reaction of ^3^[ReC]^+^ with H_2_ commences by the barrier‐free and exothermic formation of the encounter complex ^**3**^ **EC**. This step corresponds to an electrophilic attack of the positively charged metal center (*q*=+1.08) of ^3^[ReC]^+^ to the incoming ligand H_2_. Subsequently, an H atom is transferred almost barrier‐free to the carbon atom via the transition‐state ^**3**^ **TS1** (−57 kJ mol^−1^) to fall down into the rather deep well of ^**3**^ **I1**, which is located 266 kJ mol^−1^ below the reactants, and this species comprises the global minimum on the PES. The energy gained and which, given the absence of a heat bath, is stored in “hot” ^**3**^ **I1**, is almost completely reused when the latter decomposes to ^4^[ReCH]^+^ by releasing a hydrogen atom. This step is rate‐determining in the almost thermoneutral cleavage of the H−H bond by ^3^[ReC]^+^.

The proposed mechanism also fits quite well with the results of the labelling experiments: According to Figure 1 d, when ^3^[ReC]^+^ reacts with HD, formation of ReCD^+^ is favored over that of ReCH^+^. If only the conservation of angular momentum were to play a role and no kinetic isotope effects are operative, an opposite ratio of 2.0 should result in favor of ReCH^+^. However, based on the calculations, given the differences in zero‐point vibrational energies (ZPVEs), ^4^[ReCD]^+^ is by 6 kJ mol^−1^ more stable than ^4^[ReCH]^+^. Thus, the product ratio ^4^[ReCD]^+^/^4^[ReCH]^+^ should be greater than 1 (Figure 1 d).^23^

The course of the reaction of the quintet state of ReC^+^ with H_2_ is characterized by a direct mechanism, path B (blue) in Figure 4. Initially, interaction of H_2_ with the carbon atom of ^5^[ReC]^+^ leads to the formation of the very flexible and loosely bound van der Waals encounter complex ^**5**^ **EC**, which then decomposes barrier‐free by the liberation of a hydrogen atom via ^**5**^ **TS1**. The computed PES in the ^**5**^ **EC/^5^TS1** area is rather flat, and the obtained stationary points are highly approximate. Obviously, a true transition state (close or similar to ^**5**^ **TS1**) should lie somewhat above the adjacent minimum, that is, close or similar to ^**5**^ **EC** (for further details, see the Supporting Information).

In ^**5**^ **TS1**, the four atoms assemble in a collinear fashion. In general, such a geometric feature is typical for a classical hydrogen atom transfer.14b, 24 If so, there should be a three‐center/three‐electron bond (3c/3e) in ^**5**^ **TS1** involving the carbon and the two hydrogen atoms, and the bond should be featured by a doubly occupied σ_C‐H‐H_‐ and a singly occupied σ*_C‐H‐H_‐orbital.14b, 14e, 24b However, a frontier‐orbital analysis of this process does not verify this hypothesis simply because of the absence of a singly occupied σ*_C‐H‐H_‐orbital (see Figure S4b). Rather, the orbital occupancy evolution diagram (see Figure S4b) reveals a reaction mechanism which features the electronic structure requirement of a proton‐coupled electron transfer (PECT).14b, 14d, 14e Herein, the doubly occupied σ_C‐H‐H_‐orbital and the two singly occupied σ_Re‐C‐H‐H_‐ and σ*_Re‐C‐H‐H_‐orbitals form a 4c/4e bond. We note that this feature of ^**5**^ **TS1** is rather distinct from the bonding situation discovered in the [M−O]^+^/CH_4_ and [M−O]^+^/H_2_O couples, in which PCET transition states are characterized structurally by a four‐membered ring and the four electrons originate from two occupied orbitals.14b, 14d, 14e, 14g, 25

In any case, as both the intermediate and the transition state on the quintet surface are located below the separated educts ^5^[ReC]^+^/H_2_, ^5^[ReC]^+^, once generated, activates dihydrogen at ambient temperature. When HD acts as a partner for ^5^[ReC]^+^ to yield the products ^4^[ReCH]^+^ and ^4^[ReCD]^+^, the connectivity [Re−C⋅⋅⋅H⋅⋅⋅D] in ^**5**^ **TS1** is lower in energy 1.4 kJ mol^−1^ than that of [Re−C⋅⋅⋅D⋅⋅⋅H], thus slightly favoring the generation of ^4^[ReCH]^+^.^26^ However, because of the time‐dependent contributions of two processes with different preferences for the abstractions of D and H, the [ReCD]^+^/[ReCH]^+^ ratio will change with the temporal variation of the ^3^[ReC]^+^ versus ^5^[ReC]^+^ abundances. As mentioned above, this ratio will increase at longer reaction times where the reactivity is dominated by ^3^[ReC]^+^.

Path C (red) in Figure 4 illustrates the intermediates and transition state for the reaction of the singlet state ^1^[ReC]^+^ with dihydrogen. The association of H_2_ with ^1^[ReC]^+^ to form the encounter complex ^**1**^ **EC** is fairly exothermic by 180 kJ mol^−1^. In ^**1**^ **EC**, the calculated Löwdin bond order^27^ of the H−H bond amounts to 0.11, whereas a value of 0.79 is obtained for the corresponding two Re−H bonds. Obviously, in ^**1**^ **EC** the H–H σ‐bond is already significantly weakened while the metal center establishes bonds to both hydrogen atoms. Next, ^**1**^ **EC** could surmount the transition state ^**1**^ **TS1** to form the intermediate ^**1**^ **I1**. ^**1**^ **TS1** geometrically resembles ^**3**^ **TS1**, except that the former possesses a much shorter C−H and a longer H−H bond. Finally, the product ion ^2^[ReCH]^+^ could be generated by the release of an H^•^ radical in going from ^**1**^ **I1** to ^**1**^ **P1**. However, as these products are 13 kJ mol^−1^ higher in energy than the starting material, this path should not be observed under thermal conditions. Further, although we did not succeed to locate a minimal energy crossing point at the CASSCF level of theory, a conventional two‐state reactivity scenario with efficient transitions between different spin states is not likely to be operative.^28^ This is due to the fact that the singlet and the quintet surfaces are not connected by any crossing point, except for the bare ReC^+^ species on the educt side. Also for the singlet/triplet surfaces, shown in Figure 4, we did not locate any crossing points. Thus, we are left with two routes for H_2_ activation, that is, the separated reactions of the isolated triplet and quintet state of ReC^+^.

Finally, the question is addressed as to which of the two electronic states of ReC^+^ can be assigned to the faster and which to the slower reaction components (Figure 2). While the transition state for the H−H bond scission lies much lower than the separated reactants in path A for the ^3^[ReC]^+^ ground state, a significant structural rearrangement, a tight transition state, as well as substantial bond‐energy redistribution are necessary to finally liberate a H atom from H_2_. Taking further into account that on the triplet surface the overall process is almost thermoneutral, the rate efficiency should be quite sluggish for the ^3^[ReC]^+^/H_2_ couple. In contrast, for the ^5^[ReC]^+^/H_2_ system the reaction is not only much more exothermic (−80 kJ mol^−1^) but also proceeds without encountering any significant, structurally demanding barriers along the reaction coordinate. Clearly, these features are beneficial for the direct H‐atom transfer. Therefore, we conclude that it is the quintet state ^5^[ReC]^+^ which is responsible for the faster component shown in Figure 2.

In conclusion, in this combined experimental/computational work we describe the rare, if not unprecedented case for the coexistence of an electronic ground and a long‐lived, isolated excited state in a diatomic ion as exemplified by bare ReC^+^. The different electronic states exhibit distinctly different reactivities towards the thermal activation of dihydrogen. Obviously, these findings add another facet to the quite complex chemistry and unexpected mechanistic scenarios of small cluster ions.

## Experimental Section

Experimental and Computational Details

The ion/molecule reactions were performed within a Spectrospin CMS 47X Fourier transform ion cyclotron resonance (FT‐ICR) mass spectrometer; its operation is described in more detail in the experimental chapter of the Supporting Information.^16^ Extensive theoretical investigations employed a multireference perturbation theory approach, that is, the *n*‐electron valence state perturbation theory (NEVPT2).^29^ Further information on the computational details are documented in the computational chapter in the Supporting Information.

## Conflict of interest

The authors declare no conflict of interest.

## Supporting information

As a service to our authors and readers, this journal provides supporting information supplied by the authors. Such materials are peer reviewed and may be re‐organized for online delivery, but are not copy‐edited or typeset. Technical support issues arising from supporting information (other than missing files) should be addressed to the authors.

Supplementary
