# Supplementary material for: On the Crucial Role of Isolated Electronic States in the Thermal Reaction of ReC+ with Dihydrogen
Source: Angew Chem Int Ed Engl. 2020 Apr 6;59(24):9370–6. doi: 10.1002/anie.202001599 (PMC7317438; doi:10.1002/anie.202001599)
Supplement: Supplementary file 1 — Supplementary [file ANIE-59-9370-s001.pdf]

Angewandte Chemie
Eine Zeitschrift der Gesellschaft Deutscher Chemiker

Supporting Information

On the Crucial Role of Isolated Electronic States in the Thermal Reaction of ReC^+ with Dihydrogen

Jilai Li, Caiyun Geng, Thomas Weiske, and Helmut Schwarz**

anie_202001599_sm_miscellaneous_information.pdf

Table of Contents

1. Experimental Details 2
2. Computational Details 3
3. Figures 6
4. Tables 13
5. Coordinates of transition state structures in Figure 4 19
6. References 20

1. Experimental Details

The ion/molecule reactions were performed in a Spectrospin CMS 47X Fourier transform ion cyclotron resonance (FT-ICR) mass spectrometer equipped with an external ion source as described elsewhere.[1] In brief, ReC^+ was generated by laser ablation of a compressed rhenium/graphite powder (1:1; molar ratio) disk using a Nd:YAG laser operating at 532 nm; helium served as a cooling and carrier gas. It is important that the helium pipe has been baked beforehand to remove impurities on the inner walls of the feeding pipes and thus significantly improve the production of ReC^+. Using a series of ion lenses, the ions were transferred into the ICR cell, which is positioned in the bore of a 7.05 T superconducting magnet. After thermalization by about 1×10^5 collisions with pulsed-in argon, the reactions of mass-selected ReC^+ were studied by introducing isotopologues of dihydrogen (H_2, HD and D_2) via leak valves at stationary pressures. A temperature of 298 K was assumed for the thermalized clusters.[1]

The rate constants have been determined following the detailed protocol documented in the PhD Thesis of K. Koszinowski.[2] Typically, the pressures are determined with an uncalibrated Bayard-Alpard ion gauge whose reading differs depending on the kind of the gas. As the concentration of the ionic reactant A^+ is small compared to the neutral substrate B, a pseudo first-order reaction can be assumed as a good approximation,

$$d[A^+]/dt = -k[A^+][B] \cong -k_{obs}[A^+], \text{ with } -k_{obs} = -k[B]$$

here k is the true bimolecular and k_{obs} represents the apparent pseudo-unimolecular rate constant. Recording a time-dependent profile of the natural logarithm of the normalized intensity of the educt ions delivers the decline of the reactant ions whose negative slope corresponds to k_{obs}. For a general procedure to determine reaction-rate constants of ions with neutrals in the diluted gas phase, see reference.[3]

Following a protocol of Beyer and Bondybey,[4] the projectile ReC^+ was intentionally kinetically excited and over a time regime up to 1000 μs exposed to a reaction with D_2. As shown in Fig. S1, up to an excitation time of ca. 50 μs there is no significant effect on the

generation of $ReCD^+$. Finally, nearly all ICR experiments were conducted and repeated several times over a period of > 7 months. The experimental results were found robust.

Based on a careful mathematical analysis of the experimental data related to kinetics, in case of the reaction of thermalized ReC^+ with H_2 these data can best be fitted by a curve like $\mathbf{I}(\boldsymbol{t}) = \boldsymbol{a_1} \cdot \mathbf{e}^{-\boldsymbol{k1} \cdot \boldsymbol{t}} + \boldsymbol{a_2} \cdot \mathbf{e}^{-\boldsymbol{k2} \cdot \boldsymbol{t}}$; note that a two-parameter function of the type $\mathbf{I}(\boldsymbol{t}) = \mathbf{a_1} \cdot \mathbf{e}^{-\mathbf{k1} \cdot \boldsymbol{t}}$ is not sufficient to model the experimental data appropriately. As an example, the following values for the parameters a_1, k_1, a_2 and k_2 in case of the reaction of ReC^+ with H_2, which have been obtained by curve fitting, are as follows:

$$a_1 = 0.156,\ k_1 = 0.788,\ a_2 = 0.844, \text{ and } k_2 = 0.0072.$$

This analysis indicates that at the beginning of the reaction the fast reacting component (a_1) amounts to 15.6% and the slow reacting component (a_2) to 84.4% of all ReC^+ species. Also, the fast component is about 110 (k_1/k_2) times more reactive than the slower one. As shown in Figure 2b, the time evolution of the two exponents for the fast and slow reactions in the reactions of thermalized ReC^+ with H_2 at a pressure of ca.1.0 $\times 10^{-8}$ mbar indicates that the component responsible for the fast reaction is almost depleted after 5 seconds.

2. Computational Details

The calculations of the electronic structures were performed with ORCA.[5]

Quite elaborate multireference (MR) calculations were conducted in this study. The state-specific complete active space self-consistent field (CASSCF)[6] approach in conjunction with the def2-TZVP+ECP basis set (abbreviated as BS1),[7] as implemented in ORCA 4, was employed to optimize the geometries. Active spaces, (10e,10o) for ReC^+ and (12e,12o) for stationary points on the potential energy surfaces, have been considered in these MR calculations; for the selection of the active space, see Figure S2. Finally, to treat dynamic correlation without the problems of intruder states or level shifts,[8] *n*-electron valence perturbation theory (NEVPT2)[9] single-point energy (SPE) calculations were performed by using the def2-QZVPP+ECP basis set (abbreviated as BS2).[7] Harmonic vibrational frequencies were computed to verify the nature of the stationary points. The minimum

structures reported in this paper show only positive eigenvalues of the Hessian matrix, whereas the transition states (TS's) exhibit one negative eigenvalue. The thermodynamic functions (ΔH) were estimated within the ideal gas, rigid-rotor, and harmonic oscillator approximations at 298 K and 1 atm.

We should mention that the barrier ^{5}**TS1** for the ^{5}**EC** → ^{3}**P** reaction is slightly negative at the NEVPT2(12e,12o)/BS2//CASSCF(12e,12o)/BS1 level of theory (Fig. 3). The presence of ^{5}**EC** on the PES is mandatory for the topological consistency of the potential energy surface at the CASSCF(12e,12o)/BS1 level of theory. As is often the case, the upside-down situation results from the zero-point vibrational energies correction. While for the process ^{3}**EC** → ^{3}**I1** process, due to the omission of (part of) dynamical correlation in the CASSCF approach, there is considerable displacement with the high-level NEVPT2 approach which can capture the dynamic correlation for instantaneous electron motions. Due to the enormous computational demands involved, geometric optimizations at the NEVPT2 level of theory were not carried out. Rather, we resorted to apply the IRCMax[10] method as suggested by Petersson and coworkers to locate the maximum (^{3}**TS1**) and minimum (^{3}**EC**) along the triplet potential energy surface at the CASSCF(12e,12o)/BS1 level of theory (Fig. 3).

We also conducted geometry optimization for ReC^{+} at the NEVPT2 level of theory in conjunction with a ZORA-def2-QZVPP basis set for carbon[7] and a SARC-ZORA-TZVPP basis set for Re (abbreviated as BS3).[11] Subsequently, state-averaged NEVPT2 calculations were performed to obtain energetically low-lying states. These results, summarized in Table S1, clearly show the lowest three electronic states, and these are the ground state (triplet, $^{3}\Sigma^{-}$), the lowest excited state (quintet, $^{5}\Sigma^{-}$), and the second excited state (singlet, $^{1}\Gamma$) with a degenerate state ($^{1}\Sigma$). When the spin-orbit coupling effects are considered, the $^{3}\Sigma^{-}$ and $^{5}\Sigma^{-}$ states split into 3 and 5 Ω components, respectively (Tables S4 and S5).

In addition, and as requested by a reviewer, we also performed extensive calculations on the lifetimes of different electronic states due to photon emission using the detailed protocol of Ågren and co-workers.[12]. In these studies, state-averaged NEVPT2 calculations were

performed by using the NEVPT2 method in conjunction with basis sets BS4 (a ZORA-def2-TZVP basis set for carbon[7] and a SARC-ZORA-TZVP basis set for Re) and BS5 (a DKH-def2-TZVP basis set for carbon and a SARC-DKH-TZVP basis set for Re).[11] For each spin state, 5, 8, and 10 roots were considered (abbreviated as SA5, SA8, and SA10), respectively. In these calculations, molecular symmetry is switched on and very tight SCF convergence was selected. These extensive results are listed in the Tables S6-S9. We should like to state that we are aware of the fact that the calculations of the oscillator strengths, and thus the lifetimes of these species in particular when transition-metals are involved, are not only difficult but also highly dependent on the method employed.[13] However, the data given in the Tables S6-S9 demonstrate that we are not dealing with a situation in which radiative stabilization is so efficient that only extremely short-lived states are present. Rather, the take-home message is that the lifetimes of the considered low-lying electronic states extend in the time regime of seconds (and beyond) in which our experiments were conducted.

3. Figures

Figure S1. Plot of the product ion intensity as a function of the excitation time of the ReC^+ parent ion prior to its reaction with D_2 at 8×10^{-9} mbar and a reaction time of 3s. X-axis refers to the excitation time (μs), and the y-axis are normalized relative ion abundances of $ReCD^+$ and Re^+, respectively.

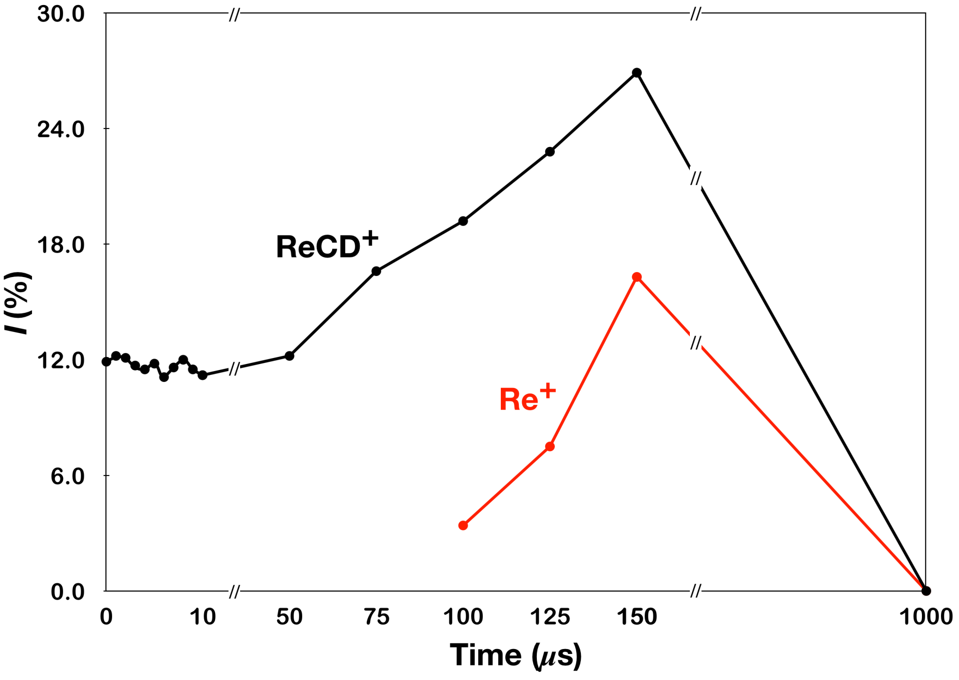

Figure S2. Selected active spaces considered in the NEVPT2(10e,10o)//CASSCF(10e,10o) calculations for (a) $^{3}[ReC]^{+}$, (b) $^{5}[ReC]^{+}$, and (c) $^{1}[ReC]^{+}$, respectively. Natural orbital partial occupation numbers are given.

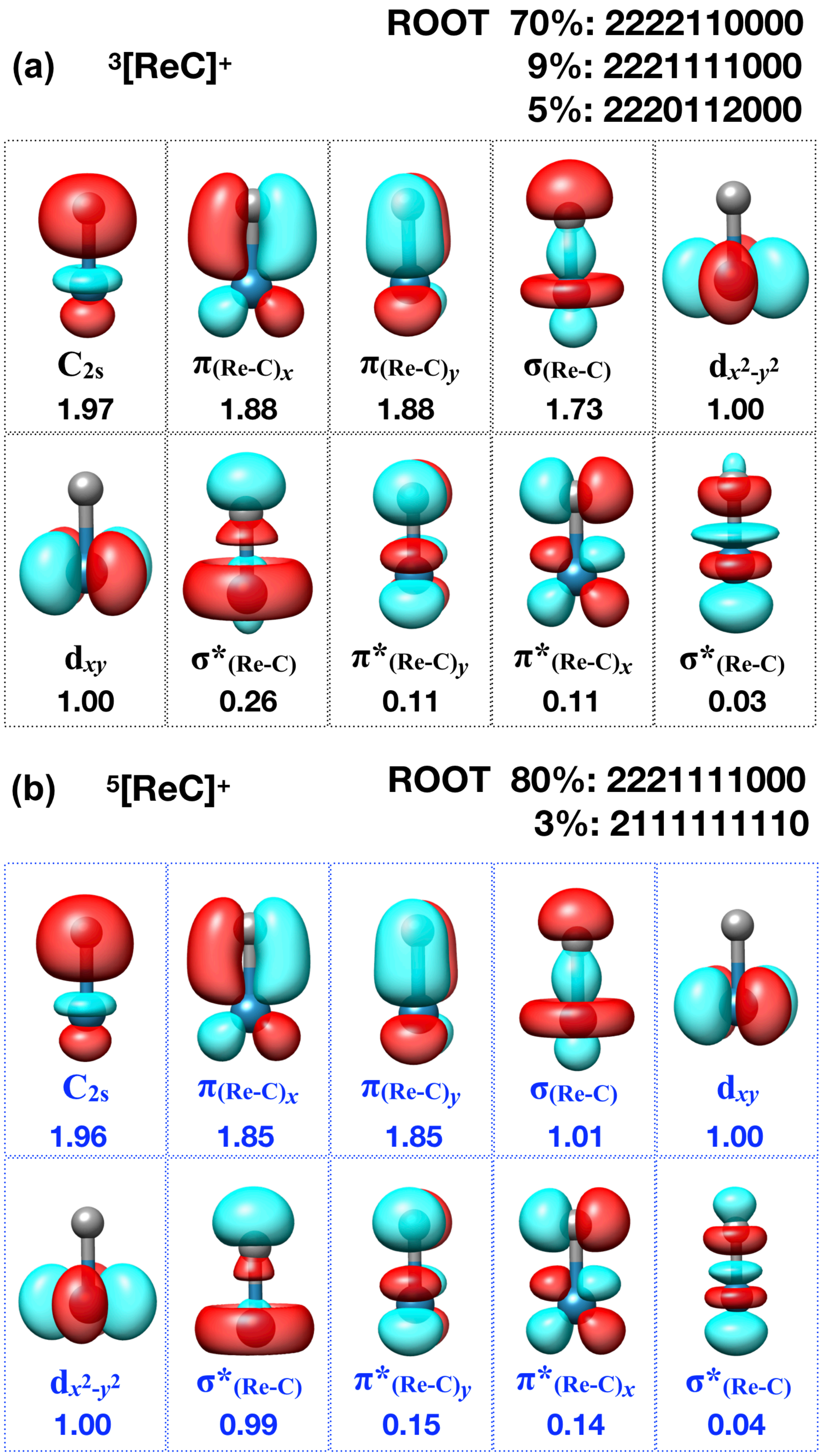

(c) $^{1}[ReC]^{+}$ ROOT 43%: 2222200000
43%: 2222020000

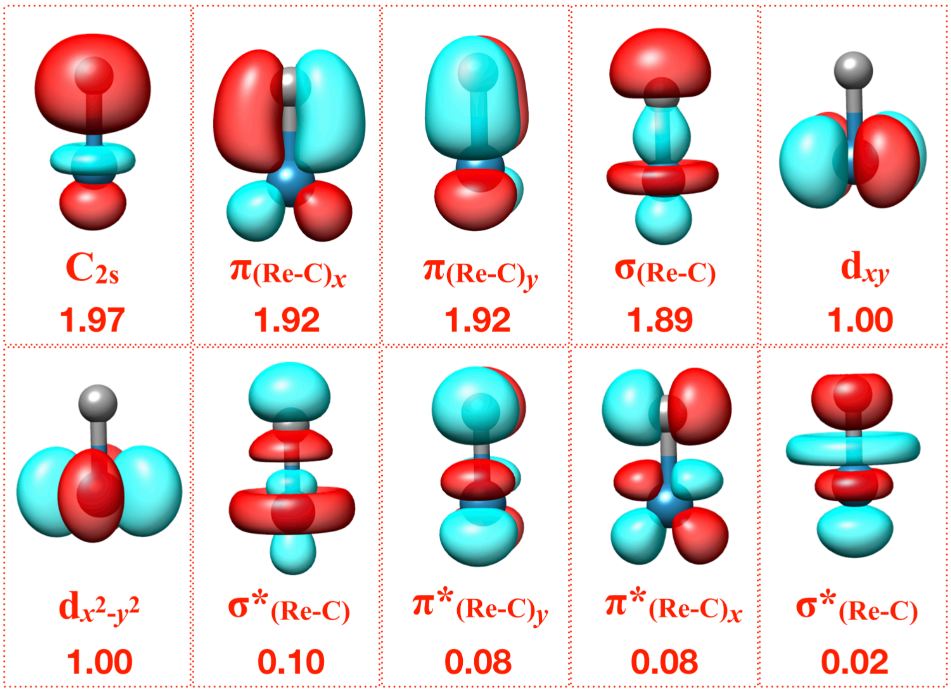

Figure S3. Alternative potential energy surface (ΔH_{298K} in kJ mol^{-1}) as obtained at the NEVPT2//CASSCF(12e,12o) level of theory for the reaction of ReC^+ with H_2. Key structures with selected geometric parameters are also provided. Bond lengths are given in Å. Charges are omitted for the sake of clarity.

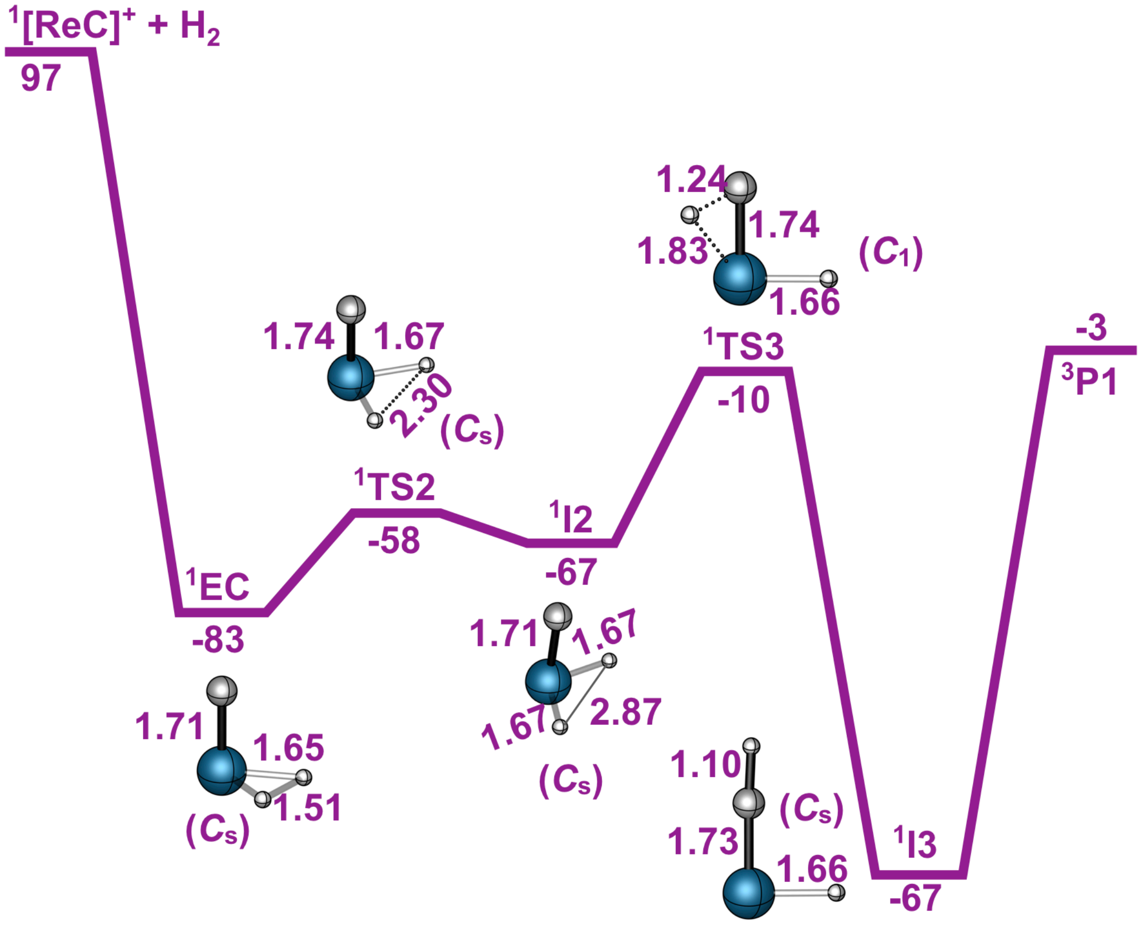

Figure S4. Schematic orbital diagrams represented by a frontier orbital analysis for selected points of the three paths A (^{3}EC→^{3}TS1→^{3}I1), B (^{5}EC→^{5}TS1), and C (^{1}EC→^{1}TS1→^{1}I1), respectively, as obtained by CASSCF(12e,12o) calculations. Natural orbital partial occupation numbers are also given.

A:

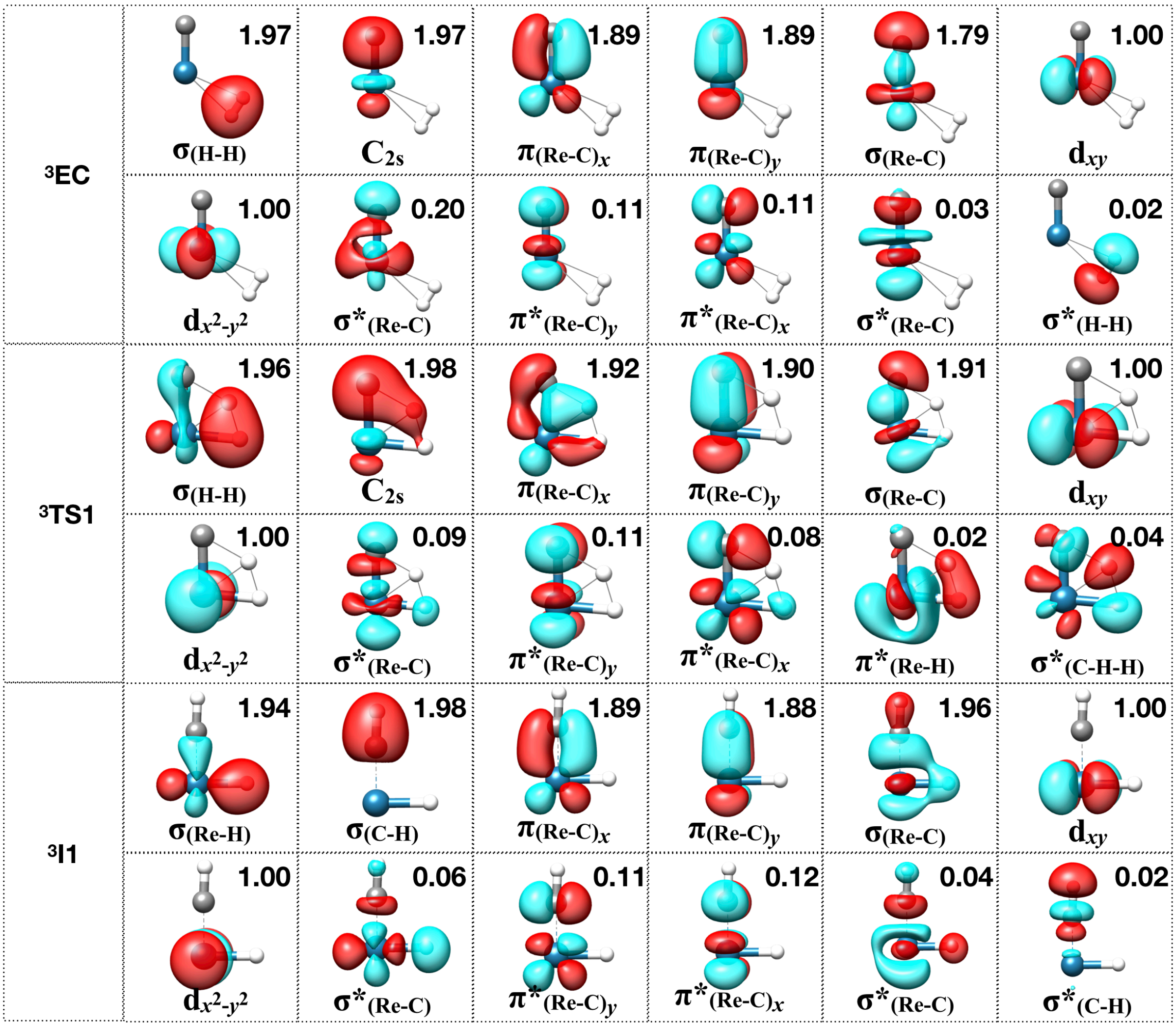

B:

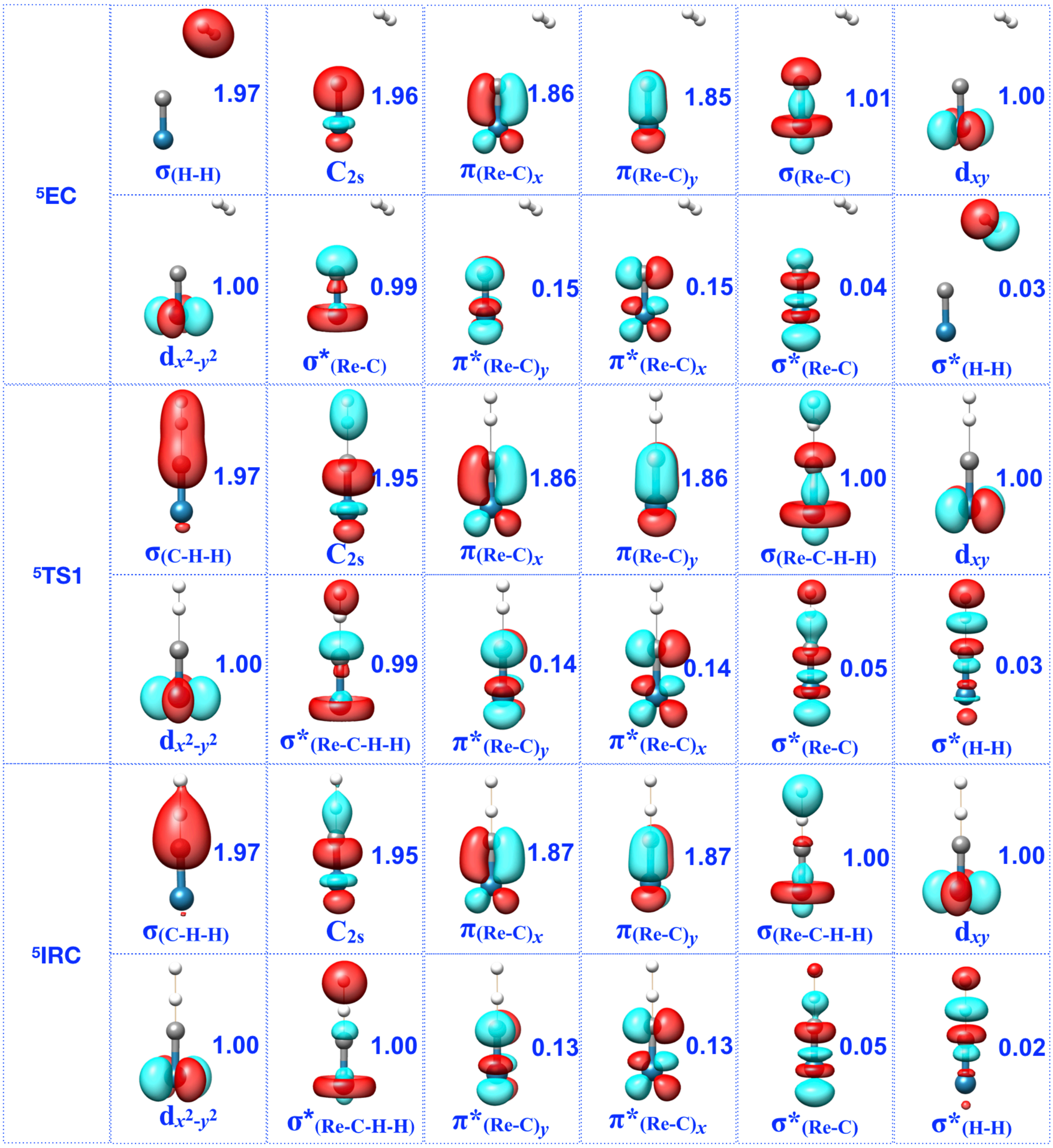

⁵EC
1.97
σ(H-H)
1.96
C2s
1.86
π(Re-C)x
1.85
π(Re-C)y
1.01
σ(Re-C)
1.00
dxy
1.00
dx²-y²
0.99
σ*(Re-C)
0.15
π*(Re-C)y
0.15
π*(Re-C)x
0.04
σ*(Re-C)
0.03
σ*(H-H)
⁵TS1
1.97
σ(C-H-H)
1.95
C2s
1.86
π(Re-C)x
1.86
π(Re-C)y
1.00
σ(Re-C-H-H)
1.00
dxy
1.00
dx²-y²
0.99
σ*(Re-C-H-H)
0.14
π*(Re-C)y
0.14
π*(Re-C)x
0.05
σ*(Re-C)
0.03
σ*(H-H)
⁵IRC
1.97
σ(C-H-H)
1.95
C2s
1.87
π(Re-C)x
1.87
π(Re-C)y
1.00
σ(Re-C-H-H)
1.00
dxy
1.00
dx²-y²
1.00
σ*(Re-C-H-H)
0.13
π*(Re-C)y
0.13
π*(Re-C)x
0.05
σ*(Re-C)
0.02
σ*(H-H)

C:

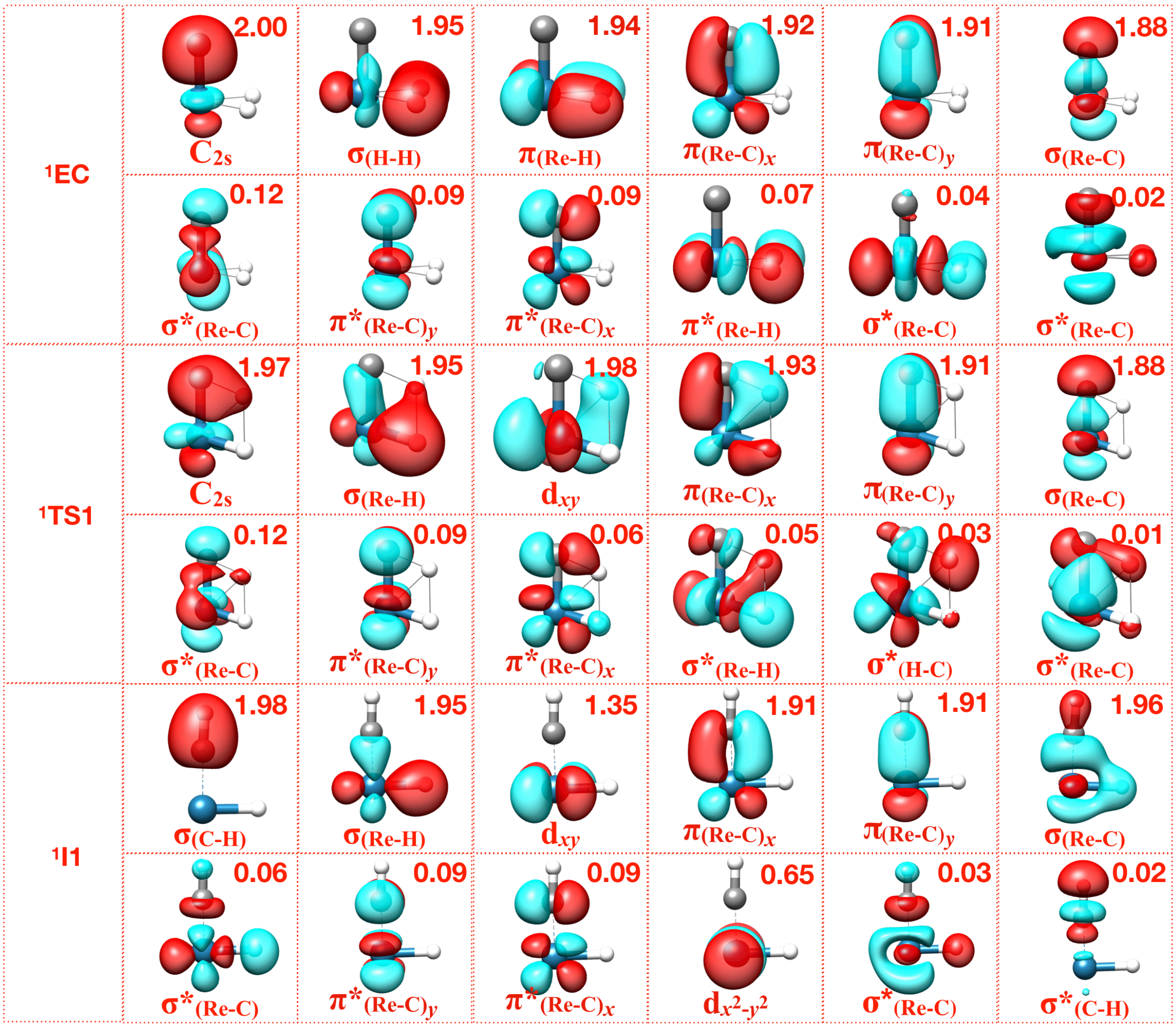

1EC
2.00
C2s
1.95
σ(H-H)
1.94
π(Re-H)
1.92
π(Re-C)x
1.91
π(Re-C)y
1.88
σ(Re-C)
0.12
σ*(Re-C)
0.09
π*(Re-C)y
0.09
π*(Re-C)x
0.07
π*(Re-H)
0.04
σ*(Re-C)
0.02
σ*(Re-C)
1TS1
1.97
C2s
1.95
σ(Re-H)
1.98
dxy
1.93
π(Re-C)x
1.91
π(Re-C)y
1.88
σ(Re-C)
0.12
σ*(Re-C)
0.09
π*(Re-C)y
0.06
π*(Re-C)x
0.05
σ*(Re-H)
0.03
σ*(H-C)
0.01
σ*(Re-C)
1I1
1.98
σ(C-H)
1.95
σ(Re-H)
1.35
dxy
1.91
π(Re-C)x
1.91
π(Re-C)y
1.96
σ(Re-C)
0.06
σ*(Re-C)
0.09
π*(Re-C)y
0.09
π*(Re-C)x
0.65
dx2-y2
0.03
σ*(Re-C)
0.02
σ*(C-H)

4. Tables

Table S1. Various states of ReC^+ as calculated at the state-averaged ZORA-NEVPT2(10e,10o)/BS3 level of theory. The relative energies (ΔE, kJ mol^{-1}) refer to the ground state.

	ΔE	Configuration	state
1	0	$1\sigma^2 1\pi^4 2\sigma^2 1\delta^2/1\sigma^2 1\pi^4 2\sigma^1 1\delta^2 3\sigma^1$	$^3\Sigma$
2	83	$1\sigma^2 1\pi^4 2\sigma^1 1\delta^2 3\sigma^1$	$^5\Sigma$
3	102	$1\sigma^2 1\pi^4 2\sigma^2 1\delta^2$	$^1\Gamma$
4	103	$1\sigma^2 1\pi^4 2\sigma^2 1\delta^2$	$^1\Sigma$
5	127	$1\sigma^2 1\pi^4 2\sigma^2 1\delta^1 3\sigma^1/1\sigma^2 1\pi^4 2\sigma^1 1\delta^3$	$^3\Delta$
6	180	$1\sigma^2 1\pi^3 2\sigma^2 1\delta^2 3\sigma^1$	$^5\Pi$
7	188	$1\sigma^2 1\pi^4 2\sigma^1 1\delta^3/1\sigma^2 1\pi^4 2\sigma^2 1\delta^1 3\sigma^1$	$^1\Delta$
8	230	$1\sigma^2 1\pi^4 2\sigma^1 1\delta^2 3\sigma^1/1\sigma^2 1\pi^4 2\sigma^2 1\delta^2$	$^3\Sigma$
9	237	$1\sigma^2 1\pi^4 2\sigma^1 1\delta^3/1\sigma^2 1\pi^4 2\sigma^2 1\delta^1 3\sigma^1$	$^3\Delta$
10	271	$1\sigma^2 1\pi^3 2\sigma^2 1\delta^3/1\sigma^2 1\pi^3 2\sigma^2 1\delta^2 3\sigma^1$	$^3\Phi/^3\Pi$
11	283	$1\sigma^2 1\pi^3 2\sigma^2 1\delta^3$	$^3\Phi$
13	308	$1\sigma^2 1\pi^4 2\sigma^2 1\delta^1 3\sigma^1/1\sigma^2 1\pi^4 2\sigma^1 1\delta^3$	$^1\Pi/^1\Delta$
12	334	$1\sigma^2 1\pi^3 2\sigma^1 1\delta^3$	$^1\Phi$
14	354	$1\sigma^2 1\pi^3 2\sigma^2 1\delta^3$	$^1\Phi$
15	401	$1\sigma^2 1\pi^3 2\sigma^1 1\delta^3 3\sigma^1$	$^5\Phi$

Table S2. Mulliken charge populations of the three lowest electronic states of ReC^+ as calculated at the CASSCF(10e,10o)/def2-TZVP+ECP level of theory.

	Charge		Spin	
	Re	C	Re	C
Triplet ($^3\Sigma^-$)	1.08	-0.08	0.00	0.00
Quintet ($^5\Sigma^-$)	1.04	-0.04	2.57	-0.57
Singlet ($^1\Gamma$)	0.94	0.06	3.47	0.53

Table S3. Bond lengths (Å) and relative energies (kJ mol^{-1}) of the three lowest electronic states of ReC^+ as obtained at two different levels of theory.

	ΔE^a	ΔE^b	R^a	R^b
Triplet ($^3\Sigma^-$)	0	0	1.698	1.658
Quintet ($^5\Sigma^-$)	77	76	1.767	1.714
Singlet ($^1\Gamma$)	96	100	1.671	1.642

[a]) calculated at the NEVPT2(10e,10o)/BS2//CASSCF(10e,10o)/BS1 level of theory.

[b]) calculated at the NEVPT2(10e,10o)/BS3 level of theory.

Table S4. The electronic states considered in the lifetime estimation due to photon emission. Data were obtained at the SA10-ZORA-NEVPT2/BS4 level of theory using the geometry calculated at the ZORA-NEVPT2/BS3 level of theory. A, triplet $^3\Sigma$ state; B, quintet $^5\Sigma$ state; C, singlet $^1\Gamma$ and $^1\Sigma$ states.

A	ΔE (cm^{-1})	Weight	Ms
$^3\Sigma$(I)	0.0	0.499	1
		0.499	-1
$^3\Sigma$(II)	31.2	0.030	1
		0.939	0
		0.030	-1
$^3\Sigma$(III)	31.2	0.469	1
		0.060	0
		0.469	-1
**B**	ΔE (cm^{-1})	Weight	Ms
$^5\Sigma$(I)	0.0	0.364	2
		0.242	0
		0.364	-2
$^5\Sigma$(II)	43.8	0.487	2
		0.487	-2
$^5\Sigma$(III)	43.9	0.487	1
		0.013	0
		0.487	-1
$^5\Sigma$(IV)	178.4	0.493	1
		0.493	-1
$^5\Sigma$(V)	179.0	0.123	2
		0.740	0
		0.123	-2
**C**	ΔE (cm^{-1})	Weight	Ms
$^1\Gamma$(I)	0.0	0.989	0
$^1\Sigma$(I)	112.6	0.997	0

Table S5. The electronic states considered in the lifetime estimation due to photon emission. Data were obtained at the SA5-DKH-NEVPT2/BS5 level of theory using the geometry calculated at the ZORA-NEVPT2/BS3 level of theory. A, triplet $^3\Sigma$ state; B, quintet $^5\Sigma$ state; C, singlet $^1\Gamma$ and $^1\Sigma$ states.

A	ΔE (cm^{-1})	Weight	Ms
$^3\Sigma$(I)	0.0	0.500	1
		0.500	-1
$^3\Sigma$(II)	0.02	0.500	1
		0.500	-1
$^3\Sigma$(III)	0.04	1.000	0
**B**	**ΔE (cm^{-1})**	**Weight**	**Ms**
$^5\Sigma$(I)	0.0	0.98	0
$^5\Sigma$(II)	30.6	0.487	2
		0.016	0
		0.487	-2
$^5\Sigma$(III)	40.5	0.487	1
		0.016	0
		0.487	-1
$^5\Sigma$(IV)	151.0	0.493	2
		0.493	-2
$^5\Sigma$(V)	151.3	0.492	2
		0.492	-2
**C**	**ΔE (cm^{-1})**	**Weight**	**Ms**
$^1\Gamma$(I)	0.0	1.000	0
$^1\Sigma$(I)	1.9	1.000	0

Table S6. The oscillator strengths (*f*) and the lifetimes (τ) for the quintet to triplet state transition. Data were obtained at the SA10-ZORA-NEVPT2/BS4, SA8-DKH-NEVPT2/BS5 (), and SA5-DKH-NEVPT2/BS5 [] levels of theory using the $^5\Sigma^-$ geometry calculated at the ZORA-NEVPT2/BS3 level of theory.

transition			ΔE (cm^{-1})	f	τ (s)
			29.5	0.00	+∞
$^3\Sigma$(I)	←	$^3\Sigma$(II)	(0.0)	(0.00)	(+∞)
			[0.0]	[0.00]	[+∞]
			29.5	0.00	+∞
$^3\Sigma$(I)	←	$^3\Sigma$(III)	(0.1)	(0.00)	(+∞)
			[0.0]	[0.00]	[+∞]
			0.0	0.00	+∞
$^3\Sigma$(II)	←	$^3\Sigma$(III)	(0.1)	(0.00)	(+∞)
			[0.0]	[0.00]	[+∞]
			5150.5	0.00	+∞
$^3\Sigma$(I)	←	$^5\Sigma$(I)	(5131.8)	(1.00×10^{-9})	(57.0)
			[5045.0]	[1.00×10^{-9}]	[58.9]
			5169.0	1.99×10^{-7}	0.3
$^3\Sigma$(I)	←	$^5\Sigma$(II)	(5167.1)	(1.90×10^{-8})	(3.0)
			[5075.6]	[0.0]	[+∞]
			5169.0	1.99×10^{-7}	0.3
$^3\Sigma$(I)	←	$^5\Sigma$(III)	(5167.2)	(0.00)	(+∞)
			[5085.5]	[0.00]	[+∞]
			5219.6	0.00	+∞
$^3\Sigma$(I)	←	$^5\Sigma$(IV)	(5277.7)	(2.00×10^{-9})	(26.9)
			[5196.0]	[2.00×10^{-9}]	[27.8]
			5219.6	0.00	+∞
$^3\Sigma$(I)	←	$^5\Sigma$(V)	(5277.7)	(2.00×10^{-9})	(26.9)
			[5196.2]	[4.00×10^{-9}]	[13.9]
			5121.0	2.10×10^{-8}	2.7
$^3\Sigma$(II)	←	$^5\Sigma$(I)	(5131.8)	(1.00×10^{-9})	(57.0)
			[5045.0]	[1.00×10^{-9}]	[58.9]
			5139.5	0.00	+∞
$^3\Sigma$(II)	←	$^5\Sigma$(II)	(5167.1)	(0.00)	(+∞)
			[5075.6]	[0.00]	[+∞]
			5139.5	2.00×10^{-9}	28.4
$^3\Sigma$(II)	←	$^5\Sigma$(III)	(5167.2)	(1.80×10^{-8})	(3.1)
			[5085.5]	[0.00]	[+∞]
			5190.1	3.55×10^{-7}	0.2
$^3\Sigma$(II)	←	$^5\Sigma$(IV)	(5277.7)	(2.00×10^{-9})	(26.9)
			[5196.0]	[4.00×10^{-9}]	[13.9]
			5190.2	3.55×10^{-7}	0.2
$^3\Sigma$(II)	←	$^5\Sigma$(V)	(5277.7)	(2.00×10^{-9})	(26.9)
			[5196.2]	[0.00]	[+∞]
			5121.0	2.10×10^{-8}	2.7
$^3\Sigma$(III)	←	$^5\Sigma$(I)	(5131.7)	(0.00)	(+∞)
			[5044.9]	[0.00]	[+∞]
			5139.5	2.00×10^{-9}	28.4
$^3\Sigma$(III)	←	$^5\Sigma$(II)	(5167.0)	(2.00×10^{-9})	(28.1)
			[5075.6]	[2.00×10^{-9}]	[29.1]
$^3\Sigma$(III)	←	$^5\Sigma$(III)	5139.5	0.00	+∞

			(5167.1)	(2.00×10^{-9})	(28.1)
			[5085.4]	[4.00×10^{-9}]	[14.5]
			5190.1	3.55×10^{-7}	0.2
$^3\Sigma$(III)	←	$^5\Sigma$(IV)	(5277.6)	(0.00)	(+∞)
			[5196.0]	[0.00]	[+∞]
			5190.2	3.55×10^{-7}	0.2
$^3\Sigma$(III)	←	$^5\Sigma$(V)	(5277.6)	(0.00)	(+∞)
			[5196.2]	[0.00]	[+∞]

Table S7. The oscillator strengths (*f*) and the lifetimes (τ) for the quintet to singlet state transition. Data were obtained at the SA10-ZORA-NEVPT2/BS4 level of theory using the $^5\Sigma^-$ geometry calculated at the ZORA-NEVPT2/BS3 level of theory.

transition			Δ*E* (cm^{-1})	*f*	τ (s)
$^5\Sigma$(I)	←	$^5\Sigma$(II)	43.8	0.00	+∞
$^5\Sigma$(I)	←	$^5\Sigma$(III)	43.9	0.00	+∞
$^5\Sigma$(I)	←	$^5\Sigma$(IV)	178.4	0.00	+∞
$^5\Sigma$(I)	←	$^5\Sigma$(V)	179.0	0.00	+∞
$^5\Sigma$(II)	←	$^5\Sigma$(III)	0.1	0.00	+∞
$^5\Sigma$(II)	←	$^5\Sigma$(IV)	134.6	1.00×10^{-09}	82794
$^5\Sigma$(II)	←	$^5\Sigma$(V)	135.1	1.00×10^{-09}	82183
$^5\Sigma$(III)	←	$^5\Sigma$(IV)	134.5	1.00×10^{-09}	82918
$^5\Sigma$(III)	←	$^5\Sigma$(V)	135.1	1.00×10^{-09}	82183
$^5\Sigma$(IV)	←	$^5\Sigma$(V)	0.6	0.00	+∞
$^5\Sigma$(I)	←	$^1\Gamma$(I)	4729.7	0.00	+∞
$^5\Sigma$(I)	←	$^1\Sigma$(I)	4851.7	0.00	+∞
$^5\Sigma$(II)	←	$^1\Gamma$(I)	4685.9	0.00	+∞
$^5\Sigma$(II)	←	$^1\Sigma$(I)	4807.9	0.00	+∞
$^5\Sigma$(III)	←	$^1\Gamma$(I)	4685.8	0.00	+∞
$^5\Sigma$(III)	←	$^1\Sigma$(I)	4807.8	0.00	+∞
$^5\Sigma$(IV)	←	$^1\Gamma$(I)	4551.3	0.00	+∞
$^5\Sigma$(IV)	←	$^1\Sigma$(I)	4673.3	0.00	+∞
$^5\Sigma$(V)	←	$^1\Gamma$(I)	4550.8	0.00	+∞
$^5\Sigma$(V)	←	$^1\Sigma$(I)	4672.8	0.00	+∞

Table S8. The oscillator strengths (*f*) and the lifetimes (τ) for the singlet to triplet state transition. Data were obtained at the SA10-ZORA-NEVPT2/BS4 level of theory using the $^1\Gamma$ geometry calculated at the ZORA-NEVPT2/BS3 level of theory.

transition			ΔE (cm^{-1})	f	τ (s)
$^3\Sigma$(I)	←	$^3\Sigma$(II)	1778.1	6.80×10^{-08}	7.0
$^3\Sigma$(I)	←	$^3\Sigma$(III)	1779.8	6.70×10^{-08}	7.1
$^3\Sigma$(II)	←	$^3\Sigma$(III)	1.7	0.00	+∞
$^3\Sigma$(I)	←	$^1\Gamma$(I)	11197.6	2.51×10^{-06}	4.76×10^{-03}
$^3\Sigma$(I)	←	$^1\Sigma$(I)	11310.2	0.00	+∞
$^3\Sigma$(II)	←	$^1\Gamma$(I)	9419.6	0.00	+∞
$^3\Sigma$(II)	←	$^1\Sigma$(I)	9532.2	0.00	+∞
$^3\Sigma$(III)	←	$^1\Gamma$(I)	9417.8	0.00	+∞
$^3\Sigma$(III)	←	$^1\Sigma$(I)	9530.4	0.00	+∞

Table S9. The oscillator strengths (*f*) and the lifetimes (τ) for the singlet to quintet state transition. Data were obtained at the SA10-ZORA-NEVPT2/BS4 level of theory using the $^1\Gamma$ geometry calculated at the ZORA-NEVPT2/BS3 level of theory.

transition			ΔE (cm^{-1})	f	τ (s)
$^5\Sigma$(I)	←	$^5\Sigma$(II)	46.9	0.00	+∞
$^5\Sigma$(I)	←	$^5\Sigma$(III)	47	0.00	+∞
$^5\Sigma$(I)	←	$^5\Sigma$(IV)	190.4	0.00	+∞
$^5\Sigma$(I)	←	$^5\Sigma$(V)	190.4	0.00	+∞
$^5\Sigma$(I)	←	$^1\Gamma$(I)	2366.3	0.00	+∞
$^5\Sigma$(I)	←	$^1\Sigma$(I)	2483.3	0.00	+∞
$^5\Sigma$(II)	←	$^5\Sigma$(III)	0.0	0.00	+∞
$^5\Sigma$(II)	←	$^5\Sigma$(IV)	143.5	1.00×10^{-09}	72843
$^5\Sigma$(II)	←	$^5\Sigma$(V)	143.5	1.00×10^{-09}	72843
$^5\Sigma$(II)	←	$^1\Gamma$(I)	2319.4	0.00	+∞
$^5\Sigma$(II)	←	$^1\Sigma$(I)	2436.4	0.00	+∞
$^5\Sigma$(III)	←	$^5\Sigma$(IV)	143.5	1.00×10^{-09}	72843
$^5\Sigma$(III)	←	$^5\Sigma$(V)	143.5	1.00×10^{-09}	72843
$^5\Sigma$(III)	←	$^1\Gamma$(I)	2319.4	0.00	+∞
$^5\Sigma$(III)	←	$^1\Sigma$(I)	2436.3	0.00	+∞
$^5\Sigma$(IV)	←	$^5\Sigma$(V)	0.0	0.00	+∞
$^5\Sigma$(IV)	←	$^1\Gamma$(I)	2175.9	0.00	+∞
$^5\Sigma$(IV)	←	$^1\Sigma$(I)	2292.9	0.00	+∞
$^5\Sigma$(V)	←	$^1\Gamma$(I)	2175.9	0.00	+∞
$^5\Sigma$(V)	←	$^1\Sigma$(I)	2292.9	0.00	+∞

5. Coordinates of the transition state structures given in Figure 4

^{3}TS1

Re	-0.16960220178690	-0.15006761243409	-0.00255829569476
C	1.51606706445117	0.17766157839766	0.00230281418306
H	0.29634158958271	1.59071262462750	0.00130331685240
H	-0.57161545224697	1.68038840940892	-0.00071483534070

^{1}TS1

Re	-0.25079880613873	0.03722150049611	0.13676744091701
C	1.42254556491428	0.33947480430478	-0.14995912509160
H	0.75530964256054	1.41464193972228	0.35332056203519
H	-0.85586540133608	1.50735675547682	-0.33979587786060

^{5}TS1

Re	-0.18680696410642	-0.19114197402486	-0.00045296433943
C	1.56124990893469	0.02582348744563	0.00102284008554
H	3.09911025701060	0.21704162441020	-0.00030604270336
H	3.92766279816111	0.31917186216902	-0.00004483304277

6. References

[1] (a) M. Engeser, T. Weiske, D. Schröder, H. Schwarz, *J. Phys. Chem. A* **2003**, *107*, 2855–2859; (b) D. Schröder, H. Schwarz, D. E. Clemmer, Y. Chen, P. B. Armentrout, V. I. Baranov, D. K. Böhme, *Int. J. Mass Spectrom.* **1997**, *161*, 175–191; (c) K. Eller, H. Schwarz, *Int. J. Mass Spectrom.* **1989**, *93*, 243–257.

[2] K. Koszinowski, PhD Thesis, D 83, Technische Universität Berlin, **2003**.

[3] A. G. Marshall, S. E. Buttrill, *J. Chem. Phys.* **1970**, *52*, 2752–2759.

[4] M. Beyer, V. E. Bondybey, *Rapid Commun. Mass. Spectrom.* **1997**, *11*, 1588-1591.

[5] F. Neese, *WIREs Comput. Mol. Sci.* **2018**, *8*, e1327.

[6] D. Hegarty, M. A. Robb, *Mol. Phys.* **1979**, *38*, 1795-1812.

[7] (a) F. Weigend, *Phys. Chem. Chem. Phys.* **2006**, *8*, 1057-1065; (b) F. Weigend, R. Ahlrichs, *Phys. Chem. Chem. Phys.* **2005**, *7*, 3297-3305.

[8] S. Guo, M. A. Watson, W. Hu, Q. Sun, G. K.-L. Chan, *J. Chem. Theory Comput.* **2016**, *12*, 1583-1591.

[9] C. Angeli, R. Cimiraglia, S. Evangelisti, T. Leininger, J. P. Malrieu, *J. Chem. Phys.* **2001**, *114*, 10252-10264.

[10] (a) G. A. Petersson, D. K. Malick, W. G. Wilson, J. W. Ochterski, J. A. Montgomery, M. J. Frisch, *J. Chem. Phys.* **1998**, *109*, 10570-10579; (b) D. K. Malick, G. A. Petersson, J. A. Montgomery, *J. Chem. Phys.* **1998**, *108*, 5704-5713; (c) M. Schwartz, P. Marshall, R. J. Berry, C. J. Ehlers, G. A. Petersson, *J. Phys. Chem. A* **1998**, *102*, 10074-10081; (d) R. T. Skodje, D. G. Truhlar, B. C. Garrett, *J. Chem. Phys.* **1982**, *77*, 5955-5976; (e) B. C. Garrett, D. G. Truhlar, R. S. Grev, A. W. Magnuson, *J. Phys. Chem.* **1980**, *84*, 1730-1748; (f) A. Kuppermann, D. G. Truhlar, *J. Am. Chem. Soc.* **1971**, *93*, 1840-1851; (g) D. G. Truhlar, *J. Chem. Phys.* **1970**, *53*, 2041-2044; (h) H. Eyring, *J. Chem. Phys.* **1935**, *3*, 107–115.

[11] (a) D. A. Pantazis, X.-Y. Chen, C. R. Landis, F. Neese, *J. Chem. Theory Comput.* **2008**, *4*, 908-919; (b) M. Bühl, C. Reimann, D. A. Pantazis, T. Bredow, F. Neese, *J. Chem. Theory Comput.* **2008**, *4*, 1449-1459.

[12] G. Baryshnikov, B. Minaev, H. Ågren, *Chem. Rev.* **2017**, *117*, 6500-6537.

[13] M. Ončák, L. Šištík, P. Slavíček, *J. Chem. Phys.* **2010**, *133*, 174303-1–174303-19.
